# The Society for Integrative Oncology Practice Recommendations for online consultation and treatment during the COVID-19 pandemic

**DOI:** 10.1007/s00520-021-06205-w

**Published:** 2021-04-14

**Authors:** Eran Ben-Arye, Channing J. Paller, Ana Maria Lopez, Shelley White, Eva Pendleton, Gunver S. Kienle, Noah Samuels, Nuria Abbawaajii, Lynda G. Balneaves

**Affiliations:** 1 Integrative Oncology Program, Lin, Zebulon, & Carmel Medical Centers, Clalit Health Services; Faculty of Medicine, Technion - Israel Institute of Technology, 35 Rothschild St, Haifa, Israel; 2 School of Medicine, Johns Hopkins University, Baltimore, MD USA; 3 Sidney Kimmel Cancer Center, Thomas Jefferson University, Philadelphia, PA USA; 4 Wellness and Integrative Health Center, Huntsman Cancer Institute, University of Utah, Salt Lake City, UT USA; 5 Memorial Sloan Kettering Cancer Center, New York, NY USA; 6 Center for Complementary Medicine, Institute for Infection Prevention and Hospital Epidemiology, Medical Center - University of Freiburg, Faculty of Medicine, Freiburg, Germany; 7 Institute for Applied Epistemology and Medical Methodology, University of Witten/Herdecke, Freiburg, Germany; 8 Center for Integrative Complementary Medicine, Shaarei Zedek Medical Center, Israel; Faculty of Medicine, Tel-Aviv University, Tel-Aviv, Israel; 9 College of Nursing, University of Manitoba, Winnipeg, MB Canada

**Keywords:** Integrative oncology, Telemedicine, Practice guidelines, Supportive care, Doctor-patient communication

## Abstract

**Objective:**

The Society for Integrative Oncology (SIO) Online Task Force was created in response to the challenges facing continuity of integrative oncology care resulting from the COVID-19 pandemic. The Task Force set out to guide integrative oncology practitioners in providing effective and safe online consultations and treatments for quality-of-life-concerns and symptom management. Online treatments include manual, acupuncture, movement, mind-body, herbal, and expressive art therapies.

**Methods:**

The SIO Online Practice Recommendations employed a four-phase consensus process: (1) literature review and discussion among an international panel of SIO members, identifying key elements essential in an integrative oncology visit; (2) development, testing, and refinement of a questionnaire defining challenges and strategies; (3) refinement input from integrative oncology experts from 19 countries; and (4) SIO Executive Committee review identifying the most high-priority challenges and strategies.

**Results:**

The SIO Online Practice Recommendations address ten challenges, providing practical suggestions for online treatment/consultation. These include overcoming unfamiliarity, addressing resistance among patients and healthcare practitioners to online consultation/treatment, exploring ethical and medical-legal aspects, solving technological issues, preparing the online treatment setting, starting the online treatment session, maintaining effective communication, promoting specific treatment effects, involving the caregiver, concluding the session, and ensuring continuity of care.

**Conclusions:**

The SIO Online Practice Recommendations are relevant for ensuring continuity of care beyond the present pandemic. They can be implemented for patients with limited accessibility to integrative oncology treatments due to geographic constraints, financial difficulties, physical disability, or an unsupportive caregiver. These recommendations require further study in practice settings.

**Supplementary Information:**

The online version contains supplementary material available at 10.1007/s00520-021-06205-w.

## Introduction

For more than two decades, oncology patients in the USA and around the world who are interested in complementary medicine have been increasingly referred by conventional healthcare professionals for integrative oncology consultation and treatment services. Integrative care approaches may include dietary/lifestyle interventions, herbal medicine, acupuncture, mind-body, manual or movement therapies, and other traditional therapies, which are offered by a range of integrative and complementary healthcare practitioners. Integrative oncology care is provided in an evidence-based manner, often within conventional oncology settings. Unlike “alternative” or “complementary” medicine, which are practiced outside or in parallel with conventional care, integrative oncology is an integral part of the multi-disciplinary and interprofessional oncology model of care that operates in conjunction with the work of oncologists, oncology nurses, psychosocial oncology practitioners, palliative care services, and other healthcare professionals. The concept of integrative oncology has been defined by Witt and colleagues as a patient-centered, evidence-informed field that “aims to optimize health, quality of life, and clinical outcomes across the cancer care continuum and to empower people to prevent cancer and to become active participants before, during, and beyond cancer treatment” [1].

In recent years, integrative oncology clinical practice has been supported by evidence arising from high-quality randomized controlled trials that indicate many integrative oncology modalities are both effective and safe for the management of issues that impinge on patients’ quality of life (QoL), such as cancer-related pain [2], chemotherapy-induced nausea and vomiting [3], cancer-related fatigue [4], and endocrine treatment-related hot flashes and night sweats [5]. The Society for Integrative Oncology (SIO), founded in 2003, has as its stated goal to advance evidence-based, comprehensive, integrative healthcare to improve the lives of people affected by cancer. The SIO is an international organization with published evidence-based guidelines for general cancer patients and for individuals living with lung and breast cancer [6, 7]. The latter guidelines were subsequently endorsed by the American Society of Clinical Oncology [8].

Integrative oncology centers have become more commonplace around the globe, with most offering therapies following a patient-centered approach. This approach involves co-designing a program of care with patients by an integrative physician or healthcare professional who is dually trained in integrative oncology and supportive cancer care [9]. The development of a patient-centered integrative oncology program requires knowledge about the effectiveness and safety of a wide range of modalities for the treatment of specific QoL-related indications. It also requires a non-judgmental approach that respects the patient’s health, beliefs and values, autonomy, and cultural background, as well as the patient’s willingness to undertake integrative oncology modalities, an important consideration for ensuring adherence to an integrative care program. In the integrative oncology setting, the physician and care team need to take into consideration the multi-modal, individualized, and dynamic nature of integrative medicine. This approach must be woven into an evidence-based, effective, safe, and feasible treatment plan that may change from visit to visit according to the patient’s evolving QoL-related concerns and the toxicities of the conventional oncology treatment.

The COVID-19 pandemic has presented a significant challenge to integrative oncology practitioners worldwide, severely limiting their ability to ensure continuity of integrative care. Several recently published commentaries discussing the impact of the COVID-19 pandemic, and associated physical distancing regulations, on cancer care provision have recommended delays in cancer treatment and a reduction in the number of in-person treatments and consultations [10–12]. Prioritization of cancer treatments by institutions during the pandemic has also meant that some treatments deemed non-essential by decision makers, include those falling under the rubric of supportive care and integrative oncology, have been made inaccessible [13]. This is particularly troubling in light of research that has shown cancer patients and survivors experiencing heightened levels of mental health issues during the pandemic exacerbated due to restricted access to timely and appropriate cancer care [14, 15]

While online consultation/treatment recommendations have been previously developed for lifestyle interventions for individuals living with cancer, they have not accounted for the challenges posed by a global pandemic [16]. More recently, clinical recommendations have been developed that have focused on the online delivery of exercise to cancer populations during the pandemic [17], and commentaries on the online provision of specific integrative oncology therapies, such as mindfulness and yoga, have emerged [18]. Pragmatic recommendations to guide integrative oncology care from a global perspective during the COVID-19 pandemic, including online patient consultation and treatment, however, are absent.

In order to address this challenge, the SIO has established the Online Task Force to share creative healthcare initiatives among integrative oncology practitioners, particularly those offering online consultations and treatment. This paper presents the SIO Online Practice Recommendations with the goal of supporting integrative oncology practitioners, including those trained in conventional and/or complementary medicine, in addressing their patients’ supportive care, QoL, and symptom management needs by providing effective and safe online integrative oncology consultations and treatments during this time of pandemic risk, which can be applied moving forward to expand the accessibility and reach of integrative oncology care worldwide even post-pandemic. For the purposes of this paper, the term “integrative oncology practitioners” refers to the variety of conventional and complementary healthcare practitioners that collaborate in the provision of integrative oncology care, including but not limited to, physicians, nurses, dietitians, psychologists, rehabilitation specialists, massage therapists, acupuncturists, and arts-based therapists.

## Methods

### Establishment of the SIO Online Task Force

The SIO Online Task Force was established in March 2020 in response to the global COVID-19 pandemic that forced many integrative oncology programs to either close or significantly limit their clinical practice. In order to meet the challenges posed by the pandemic, a number of integrative oncology practices began to develop creative online solutions to enable them to continue treating their patients without compromising their safety. News of these local healthcare initiatives quickly spread via international videoconference meetings with SIO members from Israel, Italy, Germany, Switzerland, the USA, and Canada. In turn, they shared their strategies and offered practical suggestions for providing online integrative oncology consultations and practitioner-guided treatments to patients and their caregivers. These online initiatives were then presented to the SIO Executive Committee and Board of Trustees, who agreed to the creation of the SIO Online Task Force. The goal of this group was to develop recommendations to support integrative oncology practitioners in providing evidence-based integrative oncology care to patients, caregivers, and oncology staff through online programming and resources, in the comfort and safety of their homes. The SIO Online Task Force is comprised of seven researchers, clinicians (medical doctors including oncologists, registered nurses, massage therapists, and social workers), and administrators from four countries (USA, Canada, Israel, and Germany), representing a rich multi-disciplinary, interprofessional, and multi-cultural background.

### Developing and implementing the questionnaire

Consensus methods that garner the perspectives and experiences of key stakeholders, including clinicians, administrators, and patients, have become commonplace and a standard process in the development of clinical guidelines [19]. Given the rapid evolving nature of the COVID-19 pandemic and the urgent need for recommendations that would help guide the online provision of integrative oncology consultations/treatment, an informal consensus process [20] was implemented that drew on the international expertise held within SIO and its membership. The SIO Online Practice Recommendations were developed using a four-phase consensus process led by the SIO Online Task Force. The first phase entailed a scoping review of the scientific literature conducted independently by two of the authors using PubMed, Medline, and Embase, with the goal of identifying clinical research published in the past 10 years (December 2011–December 2021) on both the efficacy and safety of online integrative oncology consultations and modalities. The following keywords were searched alone and then in combination:

- Oncology-related keywords (i.e., cancer, oncology, neoplasm, palliative, chemotherapy, radiation).
- Complementary and integrative medicine-related keywords (i.e., CAM, complementary/alternative/integrative medicine, integrative oncology, traditional medicine, herbs, herbal, mind-body, relaxation, meditation, guided imagery, hypnosis, homeopathy, acupuncture, nutritional/dietary supplements, naturopathy, energy therapy, manual therapy, massage, reflexology, yoga, qigong, Feldenkrais, anthroposophic medicine)
- Keywords related to online consultation/treatment during the COVID-19 pandemic (i.e., digital, online, online intervention, telehealth, telemedicine, virtual).

Further limits applied included articles published in English and limited to the following types of studies: clinical trials, comparative studies, meta-analyses, and systematic reviews. Practice guidelines were also included.

**Fig. 1:**
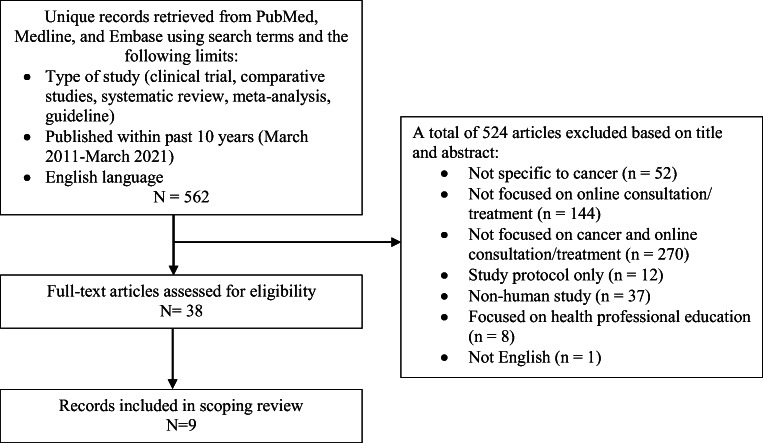
Scoping review protocol

Initially, 562 articles were identified; screening of titles and abstracts resulted in 524 articles being deemed ineligible (see Fig. 1). Of the remaining 38 articles, a review of the full text resulted in 9 articles being selected as relevant for the scoping review. Two were systematic reviews [21, 22], six were clinical trials [23–28], and one was an observational study [29]. While these articles concluded that integrative oncology therapies, such as mind-body and behavioral therapies [22, 23, 25–28], yoga [24], and exercise [21], could be feasibly offered in a virtual format and positively affect physical and psychosocial well-being, symptom management, and overall quality of life, limited pragmatic recommendations were provided regarding how to address the unique challenges of online integrative consultations/treatment. The studies examining the feasibility of online interventions provided the most useful suggestions, including the need to maintain the confidentiality of online group sessions, provide initial and on-going technical support, and address slow Internet access and limited technological knowledge [25–28]. While the online format was perceived by some patients as increasing access to care and decreasing burden [28, 29], others preferred face-to-face interactions for a more social and guided experience [22].

The findings of the scoping review, along with the clinical experience and insights of the integrative oncology teams in Israel and Europe, were presented to the SIO Online Task Force, which discussed and identified a number of core issues surrounding the provision of online integrative oncology care that required elucidation. Task Force members also identified several relevant clinical trials that assessed self-administered modalities, including self-acupressure [30], self-acupuncture [31], and self-hypnosis [32], which provided insight regarding the feasibility of self-management in the context of integrative oncology.

During the second phase of the process, the Task Force developed a questionnaire to further delineate the core issues related to providing online integrative oncology consultation and treatment modalities. A draft questionnaire was prepared and reviewed by the SIO Executive Committee and a group of leading SIO members and clinicians (physicians, nurses, psychologists, and other integrative oncology practitioners) who were actively providing online integrative oncology consultations. The final version of the questionnaire addressed three domains: (1) general challenges to providing online integrative oncology treatments, with practical suggestions for ways to overcome these challenges (25 open-ended items identified as core issues); (2) integrative oncology modalities available in integrative oncology centers before the COVID-19 pandemic, and whether they were moved online during the pandemic (16 integrative oncology modalities, provided as a checklist); and (3) specific challenges and practical suggestions associated with the provision of integrative oncology therapies via an online format (7 open-ended items that captured the main types of integrative oncology modalities). A final open-ended item was provided for respondents to share any additional insights regarding online integrative oncology consultation/treatment (see Supplementary Material for a copy of the questionnaire).

In the third phase, the final version of the questionnaire was distributed via email to 78 integrative oncology leaders from integrative oncology programs situated within leading oncology centers around the world. Some of those leaders were identified through the SIO member directory and others through snowball sampling. All were asked to share their clinical insights, with the ultimate goal of developing the SIO Online Practice Recommendation for integrative oncology care provided online. Survey replies were obtained from 54 integrative oncology experts from 19 countries (Argentina, Australia, Brazil, Canada, Chile, China, France, Germany, India, Israel, Italy, Japan, Lithuania, Netherland, Spain, Switzerland, Turkey, United Kingdom, USA) (see Table 1).

**Table 1:** Demographics of questionnaire respondents

Characteristics	*N*	%
Reported profession		
Physician (family physician, medical oncologist, pediatrician, anthroposophic physician, osteopathic doctor)	38	70.4
Nurse	2	3.7
Pharmacist	1	1.9
Psychologist	1	1.9
Administrator	7	13.0
TCM doctor	2	3.7
Naturopathic doctor	2	3.7
Homeopath	1	1.9
Gender		
Man	27	50.0
Woman	27	50.0
Geographical location		
Europe	18	33.3
Middle East	9	16.7
East Asia/South Asia	6	11.1
Australia	4	7.4
North America	12	22.2
Central and South America	5	9.3

*N* = 54

In the concluding fourth phase, the Task Force created the SIO Online Practice Recommendations after a brief thematic analysis of the open-ended survey data. This analysis was independently performed by four authors (EBA, NS, NA, LGB), who grouped the responses into a preliminary list of 10 challenges that had been identified by respondents as being associated with online integrative oncology consultations and treatments. For each of these challenges, key principles of online care were identified and specific recommendations for practical implementation were developed. This list of challenges and recommendations were thoroughly discussed by members of the SIO Online Task Force and then reviewed by the SIO Executive Committee for final confirmation. The SIO Executive Committee is an interdisciplinary group of integrative oncology key stakeholders, including oncology health professionals (i.e., physicians, nurse, psychologist), integrative oncology researchers, patient advocates, and clinical administrators. The final SIO Online Practice Recommendations presented in this article is, thus, the cumulation of a rigorous consensus process that engaged international leaders and clinicians in integrative oncology.

## Results

Table 2 presents, in detail, the SIO Online Practice Recommendations. The recommendations focus on ten challenges that need to be addressed during the online integrative oncology consultation and treatment process. Each of the ten challenges is accompanied by a list of recommendations for addressing each challenge and specific suggestions for their practical implementation. The following challenges to online integrative oncology consultations and treatments are addressed in the Online Practice Recommendations:

1. Identify any *skepticism* about, or antagonism toward, the *feasibility* and effectiveness of an online integrative oncology treatment program. Acknowledge that resistance to online consultation/treatment may not be a concern only for the patient and/or caregiver, but also for the integrative oncology practitioner.
2. Be aware of *ethical and medical-legal* issues regarding the patient’s privacy and data security, selection bias by healthcare practitioners related to which patients are offered online consultations and the treatment modalities deemed appropriate for the online format, and the potential for specific online-associated risks (e.g., breaches in patients’ privacy).
3. Assess and proactively respond to *technical challenges* associated with the virtual environment before and during an online session.
4. *Prepare the online setting* to ensure a quiet, safe, and private environment in which the therapeutic nature of the interaction can be maintained. This includes scheduling the session ahead of time; co-organizing the home setting with the patient and/or caregiver; and addressing the setting from which the integrative oncology practitioner will be conducting the online session, whether at the clinic or from home.
5. *Address potential care challenges at the beginning of the online session*, including discussing the patient’s expectations and co-defining treatment goals, while continuously monitoring the patient’s attentiveness and fatigue, especially in light of the potential for distractions due to the online remote setting.
6. Ensure effective *communication during the online session*, avoiding the use of technical and unidirectional instructions and establishing a space for patients to discuss their emotions, while emphasizing the clinical therapeutic goal, just as a practitioner would if the session were taking place in-person at a clinic.
7. Strive to promote *specific treatment effects*, such as the relief of at least one of the patient’s leading concerns (e.g., pain, fatigue, insomnia, nausea), while at the same time maximizing general and non-specific effects of the treatment (e.g., expressing compassion with regards to the patient’s sense of isolation during the pandemic).
8. Consider including the *caregiver* in the therapeutic process, based on the patient’s consent and ethical-therapeutic considerations. Clarify whether the caregiver is only there to assist the patient, or is also in need of support and treatment as well, especially in the context of therapies that are amenable to group participation, including mind-body and movement modalities (e.g., Yoga, Qi Gong, Feldenkrais method).
9. Proactively plan how to *conclude* the online integrative oncology session while ensuring a sense of containment and encouragement by scheduling a follow-up session.
10. Ensure *continuity of care* by discussing the need to schedule and coordinate the next consultation and treatment sessions, and to communicate with other integrative and conventional oncology practitioners involved in the patient’s treatment and care.

**Table 2:** Top 10 SIO Online Practice Recommendations

	Challenges to address	Principles of the recommendation	Specific suggestions for practical implementation
1	Resistance to telemedicine	1. Identify any skepticism held by the patient, health care practitioner (HCP), or caregiver toward the feasibility and effectiveness of the online treatment program 2. Develop a trusting relationship and rapport (“I understand and respect your hesitancy”) 3. Designate an integrative oncology staff member to reassure patients and caregivers and answer questions	1. Assess potential barriers to HCPs’ motivation to facilitate online treatment (e.g. “this treatment is impossible to be provided online”; “the patient is not familiar with high-tech digital media”) and criticism from colleagues concerned with undermining their professional role in the patient’s treatment (e.g., in psychotherapy, spiritual care, acupuncture, manual therapies). 2. Suggest having staff meetings to discuss these issues. 3. Assess possible barriers to the patient’s motivation to undergo online treatment (e.g., unfamiliarity with and intimidation by online technologies; skepticism regarding the effectiveness of online treatment; concerns regarding the risks due to unprofessional self-applied treatment). 4. Suggest 24/7 available support provided by the therapeutic/administrative/technical staff.
2	Ethical and medical-legal questions: risk/safety issues	1. Respect and ensure the patient’s privacy, consent 2. Maintain medical data security 3. Consider HCP selection bias for appropriate and inappropriate candidates for online treatment sessions 4. Proactively monitor risks of online treatment	1. Ensure that the online treatment is no different from conventional treatment regarding ethics (e.g., permission to film/record patients, use of shared screens, etc.) and medical file recording and protection (e.g., use of firewalls). 2. Consult a lawyer and a telehealth expert to develop procedures and communications in online platforms. 3. Develop a structured medical education training process to ensure the above ethical/safety-related issues and potential HCP referral biases (e.g., patients not able to undergo online treatment due to age and social-cultural factors or unfamiliarity with newly admitted patients). 4. Ensure a patient’s privacy; make sure the patient can speak freely, not in the presence of unwanted others (e.g., risk of abuse and domestic violence). 5. Recommend that headphones be used to ensure privacy. 6. Make sure to maintain confidentiality and avoid breaches in privacy. Patients should be placed in online “waiting rooms” to ensure only appropriate and wanted participation. 7. Conduct support groups in “closed” sessions, requiring online registration and access through private link. 8. Arrange with the institutional health network for an endorsed/secured telehealth platform. 9. Protect any transfer/sharing of the treatment session between team members (e.g., avoid social media or widely distributed e-mail). 10. Choose safe-profile interventions for the online treatment, to be supplemented by warnings, disclaimers, and rigorous clarifications that all online treatment should be performed only during the online session, under the supervision of the mentoring HCP. Have the HCP provide clear and specific instructions for self-care/exercises to be performed outside the online session.
3	Technical barriers, before and during the online session	1. Assess available technological infrastructure, connectivity and technical barriers experienced by patients using the online platform 2. Consider alternative, non-online intervention (e.g., telephone) 3. Assign a staff member to address any issues patients have accessing online platforms	1. Provide written information to patients and caregivers with step-by-step instructions on how to access the online platforms or websites 2. Check before the start of each session to ensure that it will run smoothly 3. Assess resources available to the patient (e.g., caregivers) and the institution’s technical support related to downloading apps/platforms, teaching patients how to use them, and assisting during the treatment session (in real-time) 4. Prepare the patient for switching between platforms, if needed 5. Consider providing/renting tablets with online apps for patients who have difficulty with small screens and their technical operation 6. Involve religious leaders in a dialogue about online restrictions (i.e., Sabbath); and use a caregiver to hold the cellphone (or suggest using a stand for the phone/tablet) 7. Create an independent website/YouTube channel to host videos
4	Preparing the online setting	1. Schedule the session ahead of time 2. Co-plan with the patient to ensure a quiet and safe setting with a therapeutic environment, with minimal distractions and interruptions by unwelcomed participants 3. Reserve quiet and isolated room for online treatment	1. Ensure a quiet setting for the online treatment session for both the patient and the HCP. 2. Think about creating a setting that is more “medical” for the patient (e.g., wearing a medical coat), or one with a more patient-centered focus (e.g., removing the mask at the beginning of the session).
5	Beginning the online session	1. Co-define with the patient expectations and treatment goals 2. Facilitate patients’ attentiveness, despite the online remote setting	1. Initiate the session with a brief introduction that includes technical aspects (e.g., check the sound and video quality, camera angle, disturbances, emphasis on only one person speaking at a time, using the chat box to raise questions that come up during session to be addressed at end); examine the setting (e.g., space and time); outline the agenda; and confirm the patient’s consent. 2. Co-establish with the patient (and caregiver, if invited to the session) tangible treatment goals. These may include bio-physical, emotional, and existential concerns as well as discussion on how to manage uncertainty, particularly that related to the COVID-19 pandemic. 3. Suggest a preparatory mind-body exercise (e.g., breathing, meditation) to increase awareness and focus to the subsequent treatment-related guidance.
6	Ensuring effective communication during the session	1. Emphasize that the treatment session has a clinical therapeutic goal, the same as if it were taking place in the clinic 2. Establish a space for patients to discuss their emotions and for a “being” encounter rather than a “doing” intervention 3. Assess patients’ fatigue and ability to remain focused for long online sessions, especially if the sessions are of low technical quality	1. Acknowledge the online platform tendency to focus on concrete symptoms/concerns. Open the session by asking empathically about the patient’s emotions, coping, and general well-being as well as the larger family and social context. 2. Be aware of the limited HCP-patient communication in the online setting; choose your words carefully, avoiding negative nocebo-like suggestions (e.g., use positive wording with supportive feedback rather than words like “no,” “wrong,” “don’t”). 3. Suggest an interactive approach (e.g., raising hand or use the chat button) to monitor or stop the session. 4. Slow down communication when technical issues arise. 5. Keep to the session time limit and HCP-patient therapeutic boundaries. 6. Consider shortening the session and using high-quality self-recorded supplementary videos/tapes, to be shared with the patient before, during, or following the session; or breaking up the session into an in-person period and video session. 7. Consider allowing the patient to record the session to go back for review.
7	Promoting specific treatment effects	1. Establish at least one specific outcome during the online session that the treatment can ameliorate and which is among the patient’s leading symptom/concern (e.g., anxiety, fatigue, pain, insomnia, nausea) 2. Direct treatment to achieve this aim, using HCP-guided touch, movement, and mind-body therapies. These modalities may induce both specific and non-specific effects, such as compassion, care, and sense of holistic approach in contrast with patients’ feelings of isolation and abandonment during COVID-19.	1. Select a specific integrative oncology treatment (e.g., “HCP-guided self-acupressure”) based on the following four considerations: a The treatment is evidence-based (e.g., self-acupressure in PC-6 for patients suffering from chemotherapy-induced nausea and vomiting). b The treatment is effective and safe with respect to the patient’s leading symptom/concern, as co-defined by the HCP and the patient. c The treatment is easy to demonstrate and/or provided online. d The treatment intervention is to be performed only during the online session, and under the HCP’s supervision to maintain safety and effectiveness 2. Self-demonstrate the treatment plan outline in general (e.g., gentle massage of a specific acupuncture meridian in order to induce a general sensation of warmth) followed by specific treatment details (e.g., specific point location and identification of typical De-Qi sensation of soreness and discomfort) 3. Following the HCP-guided self-location of the treatment intervention, suggest that the patient close his/her eyes, focus on their breathing, while “releasing” any performance anxiety and need for “doing” and rather encourage them “be with themselves,” waiting for a specific sensation while pressing lightly on the acupressure point. 4. Prepare a very short video (30–60 s) and leaflets with drawings and explanations in order to demonstrate specific interventions (e.g., point location during HCP-guided self-acupressure).
8	Involving the caregiver	1. Ask patients about the expectations of their caregivers, as well as their role in the patient’s care (e.g. technical or therapeutic context) 2. Clarify whether the caregiver is considered an independent client, who requires treatment as well as the patient	1. Advise the patient to get technical assistance from the caregiver (e.g., facilitating online App use, holding the cellphone during the treatment session). 2. Consider launching an online training session for caregivers for technical and/or emotional aspects of the treatment, as well as specific skills (e.g., self-acupressure, touch and movement modalities) to be guided by the HCP during the online treatment with the patient. 3. With the patient’s consent, invite the caregiver to participate in the treatment intervention (e.g., joining a guided imagery session) 4. Suggest that the caregiver film the patient’s self-care at home in order to help verify the accuracy of the treatment
9	Concluding the session	1. Plan the conclusion of the session, ensuring a sense of containment and inspiration and scheduling a follow-up session	1. Be aware that patients often experience online treatment as intense and technical, and may feel overwhelmed by the experience. Check with the patient whether any concerns are unmet, and whether the intervention is clear and understood. Prepare concisely written recommendations online. 2. Ask for the patient’s feedback to and reflection on the treatment intervention, including uncomfortable experiences. 3. Establish eye contact for at least a moment before leaving the session; consider concluding with a non-verbal “therapeutic ceremony,” or with positive suggestions like “return to the here and now,” providing a suggestion that the therapeutic effect will continue to impact the patient’s well-being.
10	Ensuring continuity of care	1. Appoint a case manager for scheduling and coordinating the next treatment sessions, and for communicating with other HCPs practicing additional treatment modalities	1. From day 1 (the initial intake at the oncology center), present the online treatment program to the patient as an option whose goal is to maintain continuity of care in addition to regular visits. 2. Ask patients about their expectations for the next meeting (e.g., frequency of online sessions). 3. Schedule the next session date, either online or in person, to take place on the same day as the conventional oncology treatment at the medical center. 4. Co-plan self-care/management interventions with the patient. 5. Provide 24/7 access to patient-case manager communication. 6. Encourage the patient to provide feedback following the session in order to identify barriers to be addressed at the next session. 7. Share the online treatment goals and outcomes with the entire multi-disciplinary oncology healthcare team to ensure effective team collaboration.

*HCP*, healthcare practitioner

## Discussion

The SIO Online Practice Recommendations for online treatment and care were created in response to the COVID-19 pandemic and are based on the insights and experience of an international group of experts dedicated to ensuring continuity of integrative oncology care despite the challenges posed by this global threat. Earlier guidance on the use of online consultation/treatment can be seen in the World Health Organization (WHO) 2019 Guideline recommendations on digital interventions for health system strengthening and the 2018 WHO Guideline on the integration of palliative care in response to humanitarian emergencies and crises [33, 34]. The 2018 WHO Guidelines recommend the use of telemedicine “under the condition that it complements, rather than replaces, face-to-face delivery of health services; and in settings where patient safety, privacy, traceability, accountability and security can be monitored.” (p. 20). The emphasis on a complementary rather than alternative role of online consultation/treatment is important and addresses the recommendation that it be implemented after careful consideration of “what can and cannot be done in the remote consultation.” (p. 21). Fortunately, many of the therapies utilized in integrative oncology are amenable to virtual presentation.

Another organization that has raised awareness regarding the use of online consultation/treatment during the COVID-19 pandemic is the European Society of Medical Oncology (ESMO). The ESMO recommendations for patients with lung cancer suggest that all non-priority out-patient appointments be converted to a telemedicine platform, acknowledging this option as a valuable tool while at the same time emphasizing that it should not completely replace standard practice [35]. Suggestions on how to optimize the use of online consultation/treatment in the oncology setting have also been published by various international researchers in the field of palliative care [10, 36, 37]. Recently, Israeli researchers reported on a qualitative study summarizing practical suggestions on how to perform online treatments in the integrative oncology setting during the COVID-19 pandemic [38].

The SIO Online Practice Recommendations for online consultations/treatment address challenges associated with telemedicine that may be different from those faced in the conventional medical setting. In the integrative oncology setting, the therapeutic relationship is reliant on verbal and non-verbal communication, and often involves hands-on treatments with the integrative oncology practitioner guiding the patient on the self-application of a therapy, such as self-acupuncture/acupressure, manual/movement modalities, and mind-body therapies. It is with these factors in mind that the SIO Online Practice Recommendations were established, with the goals of providing integrative oncology practitioners with the knowledge and skills to: (1) overcome the challenges faced in the online setting; (2) properly plan and schedule the current intervention and plan subsequent interactions; (3) monitor safety-related issues; (4) ensure ethical and open communication with patients and/or caregivers; and (5) document the online integrative oncology intervention and its outcomes.

The SIO Online Practice Recommendations and the process through which they were created have been limited by a number of factors. As with other guidelines and recommendations published during the COVID-19 pandemic, the SIO Online Practice Recommendations were created in a short period of time and in response to an unexpected worldwide crisis faced by nearly all fields of healthcare, including oncology. However, the provision of integrative oncology presented additional challenges, especially when many integrative oncology services were either closed or operating with significantly restricted activity, including the cancellation of all group-based therapies due to physical distancing restrictions. The Online Practice Recommendations were also based on expert opinions of a select group of global leaders of integrative oncology services, and require confirmatory research. As such, the present SIO Online Practice Recommendations should be seen as a preliminary step, to be followed by mixed method research that explores the feasibility, effectiveness, and safety of the Recommendations across individual and group interventions, as well as among complementary healthcare practitioners and professional associations. A revised version of the Recommendations would then be developed and could be subsequently updated, as required, and subject to a formal critical appraisal process, such as the application of the AGREE II tool [39]. The final version of the Recommendations would then be shared within the international integrative oncology community through SIO as well as with conventional oncology healthcare professionals.

From an equity, diversity and inclusion perspective, online integrative oncology care may improve access to some individuals who are unable to attend sessions in person due to a variety of economic and social reasons. However, the lack of recognition within the present SIO Online Practice Recommendations regarding the unique challenges posed by online care for those living with accessibility issues, such as hearing and visual impairment, is a limitation that requires attention. Future version of the Recommendations would benefit from the insights from other established telemedicine guidelines that have offered suggestions regarding appropriate accommodations and modifications for those living with accessibility needs [40–42].

It is of utmost importance that the SIO Online Practice Recommendations not be seen only as a local and specific initiative in response to the COVID-19 pandemic. The use of online consultation/treatment is increasingly becoming a part of conventional medical practice, and those involved in the provision of integrative oncology care should not let themselves be left behind. The guidelines are, and will continue to be, relevant to other situations, including maintaining continuity of oncology care for patients with limited accessibility to the treatment center due to factors such as geographical distance or economic constraints; patients with reduced mobility, especially during active oncology treatment or with progression of disease; and patients whose caregivers are unable to facilitate and/or support their access to the integrative oncology care setting. At the same time, it is important to emphasize that online treatments should not become a substitute for face-to-face interactions. The integration of in-person and online interactions will provide patients with a richer repertoire of integrative oncology services access, and allow for tailoring treatments to patients’ needs, expectations, and health beliefs.

## Supplementary Information

ESM 1 (DOCX 24 kb).

## Data Availability

Available on request.

## Code availability

Available on request.
