# Supplementary material for: The Society for Integrative Oncology Practice Recommendations for online consultation and treatment during the COVID-19 pandemic
Source: Support Care Cancer. 2021 Apr 14;29(10):6155–65. doi: 10.1007/s00520-021-06205-w (PMC8044504; doi:10.1007/s00520-021-06205-w)
Supplement: Supplementary file 1 — (DOCX 24 kb). [file 520_2021_6205_MOESM1_ESM.docx]

Supplementary Material - Study Questionnaire

1. Core Issues: Identifying Challenges, Suggesting Practical Solutions

Please share your insights regarding the challenges related to the following core issues in providing online integrative oncology modalities and care, as well as offering practical suggestions for resolving each one. Feel free to address any additional core issues that you feel to be relevant. If you perceive no challenges, please indicate Not Applicable (NA).

| Practical Suggestions | Challenges | Core Issue |
| --- | --- | --- |
|  |  | General |
|  |  | Ethics (i.e., confidentiality) |
|  |  | Legal risks/Safety of online IO interventions |
|  |  | How to use existing online tools |
|  |  | Sliding scale or reduced fees for low income patient |
|  |  | Financial model / Reimbursement |
|  |  | Staffing under hospital emergency plan |
|  |  | Involving the caregiver |
|  |  | Involving other IO team members |
|  |  | Ensuring continuity of IO care (e.g. weekly treatment) |
|  |  | Involvement of conventional oncology practitioners |
|  |  | Other general issues |
|  |  | IO Consultation (communication-related aspects) |
|  |  | Scheduling the consultation |
|  |  | Beginning the IO consultation |
|  |  | Facilitating IO practitioner-patient communication in the online setting |
|  |  | Concluding the IO consultation |
|  |  | Reporting/Recording IO interaction (in the patient’s medical file) |
|  |  | Economic issues (billing, etc.) |
|  |  | Other IO consultation issues |
|  |  | Technical–related issues |
|  |  | Availability of computer/tablet/smart phone, online tools and sufficient Internet connection |
|  |  | Patients who are unfamiliar with or intimidated by online interactions |
|  |  | Use of “real-time” technologies for training (e.g., location of specific acupressure points) |
|  |  | Documentation (i.e., filming and recording) of the IO interaction |
|  |  | Ensuring data security |
|  |  | Distribution of additional material related to the IO therapeutic process (articles, drawings/photos) |
|  |  | Other technical-related issues |

2. Integrative oncology (IO) therapies available before and during the COVID-19 pandemic.

We’d like to understand in greater detail what IO therapies were being offered by your program prior to COVID-19 as well as if and how they have been translated into a virtual format and the associated challenges and practical suggestions.

First, please indicate with an “X” which of the following IO therapies were offered by your program before the COVID-19 pandemic occurred:

| Offered online during COVID-19 | Available before COVID-19 | IO Therapies |
| --- | --- | --- |
|  |  | Creative arts (arts/crafts/drama, writer-in-residence, writing groups) |
|  |  | Music therapy (individual and group) |
|  |  | Meditation/mindfulness therapies (MBSR, guided imagery, meditation) |
|  |  | Exercise classes (core & stretch, resistance training, high intensity interval training) |
|  |  | Yoga |
|  |  | Pilates |
|  |  | Tai chi/Qigong |
|  |  | Dance classes (Zumba) |
|  |  | Guidance on vitamin and mineral/herbal/other supplements |
|  |  | Nutrition/dietitian consultations (weight management, cooking classes) |
|  |  | Patient/caregiver support groups |
|  |  | Consultations with psychology/social work as part of IO care (Individual, couples, family, and group therapy) |
|  |  | Hydrotherapy |
|  |  | Touch therapies (therapeutic touch, healing touch) |
|  |  | Spiritual care |
|  |  | Self-acupuncture/self-acupressure |
|  |  | Other IO therapies |

3. Challenges offering integrative oncology (IO) therapies in an online format

Please identify the challenges in offering the following IO therapies in an online format and any practical suggestions you may have to resolve these issues.

| Practical Suggestions | Challenges | Online IO Therapies |
| --- | --- | --- |
|  |  | Self-acupuncture/self-acupressure |
|  |  | Touch therapies (therapeutic touch, healing touch) |
|  |  | Mind-body-spirit therapies (meditation, guided imagery, MBSR, spiritual care) |
|  |  | Movement therapies (yoga, tai chi/qigong, pilates, dance classes) |
|  |  | Creative/expressive arts therapies (music therapy, plastic art therapy, Anthroposophic eurythmy, arts/crafts/drama, writer-in-residence, writing groups) |
|  |  | Consultation on dietary and herbal supplements & nutrition |
|  |  | Other IO therapies |

If you have any additional comments you would like to share regarding your experiences in providing IO modalities and care during the COVID-19 pandemic and how the SIO Online Task Force can assist, please share below:

_______________________________________________________________________________________________________________________________________________________________________________________________________________________________________________________________

**Supplementary Table 1. Integrative Oncology Therapies Offered Before and During (Online) the COVID-19 Pandemic**

| Therapies | Before (%) | During (Online) (%) |
| --- | --- | --- |
| Creative Arts | 21 (38.9) | 11 (20.4) |
| Music Therapy | 18 (33.3) | 10 (18.5) |
| Mindfulness | 30 (55.6) | 27 (50.0) |
| Exercise | 25 (46.3) | 20 (37.0) |
| Yoga | 18 (33.3) | 14 (25.9) |
| Pilates | 8 (14.8) | 5 (9.3) |
| Tai Chi | 16 (29.6) | 13 (24.1) |
| Dance | 4 (7.4) | 3 (5.6) |
| Natural Product Guidance | 35 (64.8) | 28 (51.9) |
| Nutrition Counselling | 30 (50.6) | 25 (46.3) |
| Support Group | 25 (46.3) | 21 (38.9) |
| Counselling | 27 (50.0) | 22 (40.7) |
| Hydrotherapy | 1 (1.9) | 0 |
| Touch Therapies | 22 (40.7) | 8 (14.8) |
| Spiritual Therapies | 20 (37.0) | 12 (22.2) |

(N = 54)
